# Prognostic value of pretreatment serum fibrinogen in young patients with small cell lung cancer: A cross‐sectional study

**DOI:** 10.1002/hsr2.1507

**Published:** 2023-08-18

**Authors:** Juncai Tian, Huohuan Tian, Mengzhu Yang, Linhui Yang, Dan Liu

**Affiliations:** ^1^ Department of Respiratory and Critical Care Medicine West China Hospital of Sichuan University Chengdu Sichuan China

**Keywords:** overall survival, prognostic factor, serum fibrinogen, small cell lung cancer

## Abstract

**Objective:**

To explored the prognostic value of pretreatment serum fibrinogen level in young patients with small cell lung cancer (SCLC).

**Methods:**

The concentrations of serum fibrinogen of 183 young (<50 years old) SCLC patients were measured. The association of baseline serum fibrinogen level and clinical outcome was analyzed by survival analysis.

**Results:**

Hyperfibrinogenemia was significantly associated with American Veterans Administration Lung Study Group stage and surgery treatment. The survival analysis indicated that patients with hypefibrinogenemia had worse outcome than patients with normal fibrinogen. Pretreatment serum fibrinogen level was identified as a worse independent survival predictor in young SCLC. The hazard ratio was 1.420 (95% confidence interval: 1.035−1.947).

**Conclusions:**

Pretreatment serum fibrinogen is independent associated with overall survival in patients with young SCLC.

## Key points

- Serum ﬁbrinogen level was inversely correlated with overall survival of young small cell lung cancer (SCLC).
- Serum ﬁbrinogen concentration was signiﬁcantly associated with American Veterans Administration Lung Study Group stage.
- Pretreatment serum ﬁbrinogen was an independent prognostic factor in young SCLC.

## 1 INTRODUCTION

Lung cancer is the leading cause of cancer‐related death in the world.^1^ Small cell lung cancer (SCLC) accounts for approximately 15% of all lung cancers, and 60% of cases present with extensive‐stage (ES) disease.^2^ SCLC is prone to metastasis widespread. While immunotherapy has been added as a treatment option in recent years, the prognosis of SCLC remains poor.^3^ Accurate prognosis prediction for SCLC is crucial for the personalized treatment and medical management. Several laboratory variables have been founded as prognostic indicators for SCLC, such as serum pro‐gastrin‐releasing peptide (ProGRP), neuron‐specifific enolase, and so on.

Fibrinogen is a pivotal element of the coagulation system, which plays a major role in regulating blood clotting, inflammation, and neoplasia.^4^ Increased activity was associated with cancer cell growth and progression.^5^ Hyperfibrinogenemia is related to malignancies, such as colorectal,^6^ gastric,^7^ and non‐small cell lung cancer (NSCLC).^8^ However, the prognostic value of pretreatment serum fibrinogen in SCLC remains unknown.

SCLC in young patients has unique socioeconomic and clinical implications.^9^ Very few studies have described SCLC in young (<50 years old) patients.^9^, ^10^ The study aims to explore the impact of baseline serum fibrinogen on clinical outcome in young patients with SCLC. We hypothesize that pretreatment serum fibrinogen concentration is related to overall survival (OS) of young patients with SCLC.

## 2 METHODS

### 2.1 Study design and population

Our institutional review board approved this retrospective study and waived informed consent. The study was conducted at West China Hospital of Sichuan University, China. All subjects were newly pathologically confirmed to have SCLC between January 2014 and October 2018. The data of 820 SCLC patients were extracted by our hospital's database. There were 208 young SCLC patients. Of these patients, 14 patients were excluded due to lack of data on pretreatment serum fibrinogen, 6 patients with previous cancer history, and 5 patients with liver disease or coagulation disease also were excluded. The final analysis comprised 183 eligible young SCLC patients.

### 2.2 Data collection

The electronic medical records of SCLCA patients were extracted and analyzed by the research team. Information evaluated included age at diagnosis, sex, smoking status, tumor histology, SCLC stage according to the Veteran's Administration Lung Group's 2‐stage (VALG) classification, level of baseline serum fibrinogen, albumin, triglyceride, and blood sugar, as well as treatment protocol, and survival outcome.

Follow‐up was performed at 6‐month intervals until death, or until the last date of December 31, 2022. OS was defined as the time from the date of diagnosis until death due to any cause or the date the patient was last seen alive. Serum fibrinogen concentration of ≥4.0 g/L was defined as hyperfibrinogenemia, and concentration of 2.0−4.0 g/L was considered normal.

### 2.3 Statistical analysis

Data for categorical variables were expressed as frequency rates and percentages and were compared using the *χ* ^2^ test. Continuous variables were described using mean, median, and interquartile range values, and differences between groups were compared using the Mann−Whitney *U* test. Fibrinogen concentration was analyzed as a continuous variable and a categorical variable after grouping by normal levels and hyperfibrinogenemia. Kaplan−Meier survival curves and Cox proportional hazards model (univariate and multivariate analysis) were used to analyze the OS. The analyses were performed with Statistical Package for the Social Sciences software (SPSS 26.0) and MedCalc statistical software (version 20). Differences were considered significant at *p* < 0.05 with a two‐tailed test.

## 3 RESULTS

### 3.1 Patients characteristics

As shown in Table 1, a total of 183 young SCLC patients were included in the study. The patients comprised 118 males (64.5%) and 65 females (35.5%), with a median age of 45 years (range: 42−47 years). Ninety‐six (52.5%) of the patients had smoking history. With regard to tumor stage, 71 of the cases (38.8%) were limited stage (LS) and 112 (61.2%) ES according to the VALG classification. Regarding treatment, 169 patients (92.3%) received chemotherapy, 54 (29.5%) received radiation therapy, and 24 (13.1%) received surgery.

**Table 1:** Association between pretreatment serum fibrinogen level and clinical parameters in young SCLC patients.

Parameters	Patients, *n* (%)	Fibrinogen		*p*	Fibrinogen	*p*
		Normal (2−4 g/L)	Hyperfibrinogenemia (≥4 g/L)		Media (mean, 25th−75th)	
Age (years)		43 ± 4	43 ± 5	0.634		
Sex				0.089		0.132
Female	65 (35.5)	40 (40.4%)	25 (29.8%)		3.71 (2.92−4.89)	
Male	118 (64.5)	59 (59.6%)	59 (70.2%)		4.02 (3.17−5.19)	
Smoking				0.567		0.618
No	87 (47.5)	49 (49.5%)	38 (45.2%)		3.77 (2.99−4.96)	
Yes	96 (52.5)	50 (50.5%)	46 (54.8%)		3.93 (3.04−5.21)	
Laboratory data						
Triglyceride (mmol/L)		1.31 ± 0.75	1.17 ± 0.61	0.187		
Albumin (g/L)		42.27 ± 4.59	39.63 ± 4.78	0.001		
Blood sugar (mmol/Ll)		5.11 ± 0.81	5.66 ± 1.76	0.009		
VALG stage				0.004		0.001
LS	71 (38.8)	48 (48.5%)	23 (27.4%)		3.18 (2.63−4.29)	
ES	112 (61.2)	51 (51.5%)	61 (72.6%)		4.24 (3.49−5.37)	
Chemotherapy				0.481		0.840
Yes	169 (92.3)	92 (92.9%)	77 (92.3%)		3.81 (3.0−5.11)	
No	14 (7.7)	7 (7.1%)	7 (8.3%)		4.17 (3.75−5.54)	
Radiation therapy				0.338		0.410
Yes	54 (29.5)	31 (31.3%)	23 (27.4%)		3.74 (2.92−5.02)	
No	129 (70.5)	68 (68.7%)	61 (72.6%)		3.91 (3.04−5.18)	
Surgery				0.060		0.023
Yes	24 (13.1)	17 (17.2%)	7 (8.3%)		3.16 (2.48−5.04)	
No	159 (86.9)	82 (82.8%)	77 (91.7%)		3.94 (3.12−5.12)	

*Note*: Data are expressed as the mean ± standard deviation or median (interquartile range) or *n* (%).

Abbreviations: ES, extensive stage; LS, limited stage; SCLC, small cell lung cancer; VALG, Veterans Administration Lung Study Group.

### 3.2 Correlation of serum fibrinogen level with clinical features

The median of pretreatment serum fibrinogen level was 3.85 g/L (range: 3.03–5.12 g/L) for all SCLC patients. Of 183 patients, 84 (45.9%) had hypefibrinogenemia (≥4 g/L), and 99 patients (54.1%) had normal serum fibrinogen concentration (2−4 g/L). Pretreatment serum fibrinogen level was correlated with albumin level and blood sugar level. Patients with hyperfibrinogenemia had significantly lower albumin levels (39.63 vs. 42.27 g/L, *p* < 0.001) and higher blood sugar levels (5.66 vs. 5.11 mmol/L, *p* = 0.009) than in patients with normal fibrinogen. Moreover, pretreatment serum fibrinogen level was significantly correlated with VALG stage and surgery treatment. Fibrinogen concentration was markedly higher in ES than in LS (4.24 vs. 3.18 g/L, *p* < 0.001), and in patients without surgery than in those with surgery (3.94 vs. 3.16 g/L, *p* = 0.023) (Table 1).

### 3.3 Relationship between hyperfibrinogenemia and the frequency of venous thromboembolism

Among 183 young SCLC patients, 12 patients had venous thrombosis, with an incidence rate of 6.6%. Hyperfibrinogenemia was associated with venous thrombosis in young SCLC patients. Patients with hyperfibrinogenemia had a higher incidence of venous thromboembolism when compared to patients with normal fibrinogen level (8 cases, 66.7% vs. 4 cases, 33.3%, *p* = 0.014).

### 3.4 Changes in fibrinogen of SCLC patients after treatment

**Figure 1:**
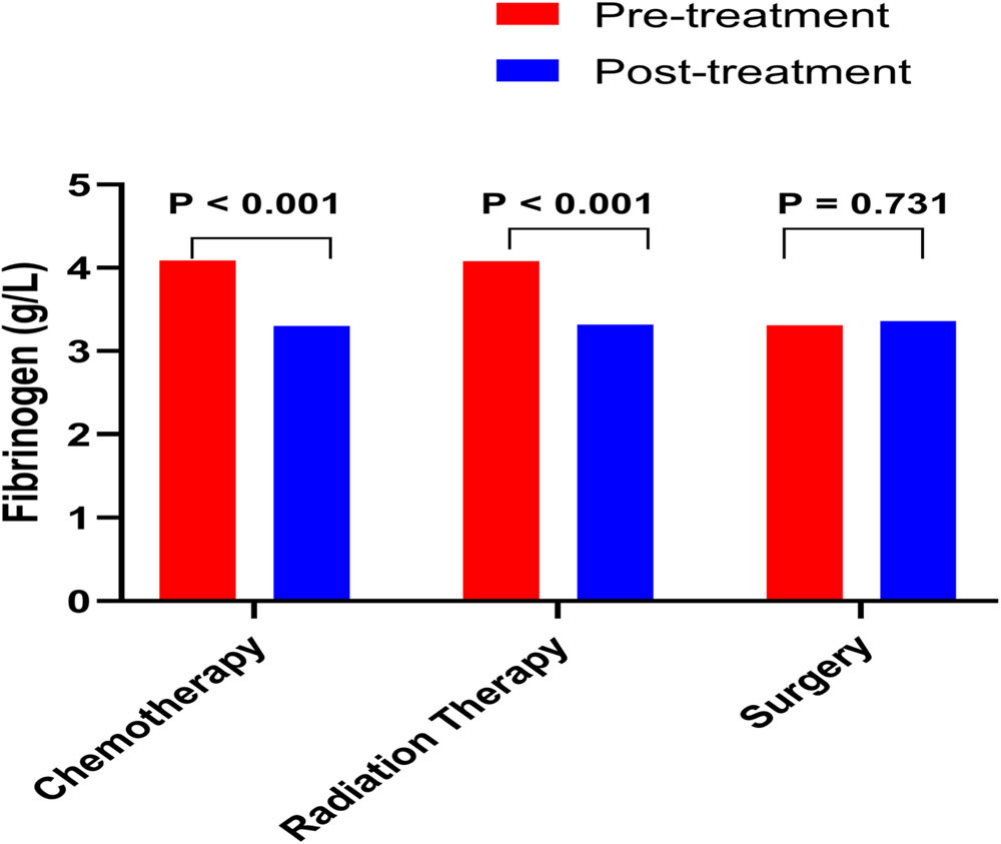
Changes in fibrinogen of SCLC patients after treatments. The fibrinogen level was significantly decreased after chemotherapy and radiation therapy (*p* < 0.001 for both). There was no significantly change in fibrinogen level after surgery (*p* = 0.731). SCLC, small cell lung cancer.

As shown in Figure 1, follow‐up fibrinogen level after the fourth treatment cycle was tested. The fibrinogen level was significantly decreased after chemotherapy and radiation therapy (*p* < 0.001 for both). There was no significantly change in fibrinogen level after surgery (*p* = 0.731).

### 3.5 Univariate and multivariate analysis of OS in SCLC

The results of univariate and multivariate cox regression analysis of OS in young SCLC patients were shown in Table 2. VALG stage, surgery treatment, and pretreatment fibrinogen level were significantly associated with OS in young SCLC by univariate analysis (*p* < 0.005 for all). LS, surgery, and low pretreatment fibrinogen level were associated with a prolonged OS. Gender, smoking history, chemotherapy, and radiotherapy were not associated with OS on regression analysis. Multivariate analysis determined that VALG stage, surgery, and pretreatment fibrinogen level were independent prognostic factors of OS (*p* < 0.005 for all). Patients with ES had an elevated risk of death compared to those with LS. The hazard ratio was 1.935 (95% confidence intervals [CI]: 1.377−2.719) for death. Patients who received surgery had an decreased risk of death compared to those without surgery treatment. The hazard ratio was 0.338 (95% CI: 0.188−0.606) for death. Patients with hyperfibrinogenemia had an elevated risk of death compared to those with normal fibrinogen level. The hazard ratio was 1.420 (95% CI: 1.035−1.947) for death.

**Table 2:** Univariate and multivariate analysis of overall survival in young SCLC patients using the Cox regression analysis.

Variables	Univariate analysis		Multivariate analysis	
	HR (95% CI)	*p* Value	HR (95% CI)	*p* Value
Age	1.005 (0.971−1.039)	0.785		
Gender (male vs. female)	1.155 (0.883−1.601)	0.387		
Smoking (yes vs. no)	1.077 (0.787−1.474)	0.644		
VALG stage (LS vs. ES)	1.882 (1.343−2.637)	<0.001	1.935 (1.377−2.719)	<0.001
Chemotherapy (yes vs. no)	0.756 (0.418−1.365)	0.353		
Radiation (yes vs. no)	0.891 (0.631−1.259)	0.513		
Surgery (yes vs. no)	0.388 (0.220−1.687)	0.001	0.338 (0.188−0.606)	0.003
Fibrinogen (normal vs. high)	1.427 (1.401−1.955)	0.027	1.420 (1.035−1.947)	0.030

Abbreviations: CI, confidence intervals; ES, extensive stage; HR, hazard ratio; SCLC, small cell lung cancer; VALG, American Veterans Administration Lung Study Group.

In addition, we performed univariate and multivariate cox analysis for exploring the relationship between pretreatment fibrinogen level and OS in the remaining 538 elderly SCLC patients with pretreatment fibrinogen values. Univariate analysis identified that pretreatment fibrinogen level was significantly associated with OS in elderly SCLC (*p* = 0.011). Multivariate analysis (the regression model included age, sex, and fibrinogen level) demonstrated that pretreatment fibrinogen level was independent prognostic factor of OS (*p* = 0.008). The hazard ratio was 1.282 (95% CI: 1.0670−1.540) for death.

### 3.6 Correlation of serum fibrinogen level with young SCLC survival

**Figure 2:**
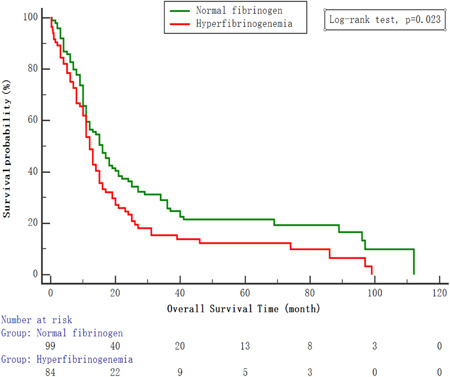
Kaplan−Meier overall survival (OS) analysis of young SCLC patients (*n* = 183). OS subdivided by serum fibrinogen level (log rank test, *p* = 0.023). SCLC, small cell lung cancer.

The median follow‐up time was 28.0 months (3−42 months). In the analysis median OS was 13.0 months (95% CI: 8.0−27.0 months). The 3‐year OS rate was 20.8%. Serum fibrinogen concentration was inversely correlated with OS. Patients with hyperfibrinogenemia had lower 3‐year OS rate (13.1% vs. 27.3%) than those with normal serum fibrinogen level. Patients with hyperfibrinogenemia had worse OS than patients with normal fibrinogen (Figure 2).

## 4 DISCUSSION

This was a retrospective study on the association between the coagulation and outcome in young patients with SCLC. As we know, this is the first study to investigate prognostic value of serum fibrinogen in young patients with SCLC. We demonstrated that pretreatment serum fibrinogen level was an independent prognostic biomarker for OS in young SCLC.

The correlation of the progression of human cancers and coagulation function has been studied for decades. The activation of fibrinolysis is generally associated with invasion, metastasis, and death. The risk of venous thrombosis is higher in NSCLC than those in cancer‐free patients.^11^ Hypercoagulability generally means more aggressive in cancer patients. Higher concentration of d‐dimer,^12^ antithrombin III content,^13^ and platelet count^14^ are associated with worse outcome in NSCLC. Moreover, high serum fibrinogen concentration is related to worse outcome in NSCLC.^15^ In this study, we found that hyperfibrinogenemia was associated with venous thrombosis in young SCLC patients. Patients with hyperfibrinogenemia had a higher risk of venous thromboembolism than those patients with normal fibrinogen level. Higher concentration of fibrinogen was associated with worse outcome in young SCLC.

Our study demonstrated that pretreatment serum fibrinogen level was correlated with albumin level and blood sugar level. Patients with hyperfibrinogenemia had significantly lower albumin levels and higher blood sugar levels than in patients with normal fibrinogen. The results suggest hyperfibrinogenemia may influence the protein and sugar metabolism in SCLC patients.

The fibrinogen level was significantly decreased after chemotherapy and radiation therapy in young SCLC. Therefore, fibrinogen level could be a tool of treatment monitoring of SCLC. However, there was no significantly change in fibrinogen level after surgery. This is due to lower baseline fibrinogen level in patients who received surgery treatment.

Surgery treatment was significantly associated with OS in young SCLC by univariate analysis. However, chemotherapy was not correlated with OS in our study. We think this result was due to the limited number of patients who was not treated with chemotherapy (*n* = 14, 7.7%).

Serum fibrinogen level was significantly correlated with VALG stage and clinical outcome in young SCLC in the study. Elevated fibrinogen level was correlated with poor OS. Cox regression analysis demonstrated that Serum fibrinogen level was independently associated with OS in young SCLC patients. Patients with hyperfibrinogenemia had 1.42 times the risk of death of those with normal fibrinogen level. Similarly, serum fibrinogen level was significantly correlated with OS in elderly SCLC population. The apparent reason for the relationship between elevated fibrinogen level and poor outcomes of SCLC remains unclear. Fibrinogen may influences many cellular processes during tumorigenesis and metastasis. Furthermore, fibrinogen layers help tumor cells block natural killer cytotoxicity with thrombin.^16^

This study had several limitations. First, the study was a single retrospective study, which may have led to unavoidable bias. Second, the sample was relatively small. Larger prospective studies are needed to confirm our results.

## 5 CONCLUSIONS

Pretreatment serum fibrinogen is a novel prognostic biomarker for OS in young SCLC. The finding could lead to a personalized treatment after diagnosis.

## AUTHOR CONTRIBUTIONS

**Juncai Tian**: Conceptualization; methodology; writing—original draft. **Huohuan Tian**: Data curation; investigation; software. **Mengzhu Yang**: Data curation; investigation; methodology. **Linhui Yang**: Conceptualization; data curation; formal analysis; methodology. **Dan Liu**: Conceptualization; supervision; writing—review and editing.

## CONFLICT OF INTEREST STATEMENT

The authors declare no conflict of interest.

## TRANSPARENCY STATEMENT

The lead author Dan Liu affirms that this manuscript is an honest, accurate, and transparent account of the study being reported; that no important aspects of the study have been omitted; and that any discrepancies from the study as planned (and, if relevant, registered) have been explained.

## Data Availability

Data of this study are available from the corresponding authors upon reasonable request.
